# Case Report: Multidisciplinary and individualized management of periprocedural arterial thrombosis and ischemic stroke in children: a three-case series

**DOI:** 10.3389/fped.2026.1967773

**Published:** 2026-10-08

**Authors:** Guoxiang Zhou, Wen Ge, Hao Wan, Rui Chen, Kefeng Hou, Zhixian Ji

**Affiliations:** 1 Heart Center, Women and Children’s Hospital, Qingdao University, Qingdao, China; 2 Department of Radiology, Women and Children’s Hospital, Qingdao University, Qingdao, China; 3 Pediatric Ultrasound Department, Women and Children’s Hospital, Qingdao University, Qingdao, China

**Keywords:** arterial thromboembolism, case report, ischemic stroke, pediatrics, thrombectomy

## Abstract

Pediatric arterial thromboembolism is rare but may cause limb loss, extensive cerebral infarction, permanent disability, or death. Evidence guiding reperfusion therapy in children remains limited. We describe three periprocedural events treated according to their vascular territory, threatened tissue, and bleeding risk. A male neonate born at 37 weeks’ gestation developed catheter-associated left external iliac–femoral arterial thrombosis after umbilical arterial catheterization during treatment for shock and respiratory illness. Intravenous urokinase and low-molecular-weight heparin achieved complete thrombus resolution on ultrasonography six days after diagnosis. A 5-year-and-5-month-old boy developed acute left internal carotid–middle cerebral artery occlusion with extensive left hemispheric infarction three days after thoracoscopic resection of pulmonary sequestration. Emergency stent-retriever thrombectomy with aspiration achieved immediate recanalization, which persisted at one week; independent ambulation was restored after rehabilitation, although mild right upper-limb symptoms remained at discharge. A 4-month-old boy developed right frontotemporal–insular ischemic infarction after balloon angioplasty for recurrent aortic coarctation. Because no retrievable intracranial large-vessel occlusion was identified and the recent cardiac intervention increased the bleeding risk, aspirin, low-molecular-weight heparin, and supportive treatment were administered. Follow-up MRI one week after diagnosis showed regression of the initial lesion but revealed a new right thalamic lesion suspicious for acute infarction. These heterogeneous cases highlight the need for prompt vascular imaging and individualized multidisciplinary management based on the vascular territory, threatened tissue, patient size, recent procedures, bleeding risk, and procedural feasibility. As a descriptive case series, this report does not establish the comparative efficacy of the treatment strategies.

## 1 Introduction

Arterial thromboembolism is substantially less common than venous thromboembolism in children, with a reported incidence of approximately 0.03 per 10,000 children (1–4). Despite its rarity, delayed recognition may result in limb ischemia, tissue loss, extensive cerebral infarction, permanent disability, or death. Risk factors include intra-arterial catheterization or intervention, congenital heart disease, surgery, critical illness, infection, trauma, prematurity, and congenital or acquired vascular abnormalities (5–7).

Evidence guiding pediatric reperfusion treatment is limited, and adult protocols cannot be transferred directly because of age-dependent differences in vessel caliber, hemostasis, drug dosing, procedural feasibility, and bleeding risk. Available options include anticoagulation, systemic or catheter-directed thrombolysis, surgical thrombectomy, and endovascular mechanical thrombectomy (5). Therefore, the decision regarding whether a child should undergo this procedure must be individualized, based on a comprehensive assessment of threatened organs, thrombus location and burden, presence of large vessel occlusion, symptom severity, time from symptom onset, recent surgical history, and procedural risks. In addition, age-related factors should also be taken into account. Ultimately, optimal management heavily relies on multidisciplinary collaborative evaluation.

This report describes three pediatric arterial thromboembolic events treated at our institution between January and June 2026 and managed with pharmacological thrombolysis, emergency mechanical thrombectomy, or anticoagulant plus antiplatelet therapy. It is intended to illustrate the clinical reasoning underlying different management choices, rather than to compare the effectiveness of different interventions directly.

## 2 Case description

### 2.1 Case 1: neonatal external iliac–femoral arterial thrombosis

A male neonate was delivered by cesarean section at 37 weeks of gestation due to fetal distress, with a birth weight of 3.32 kg and a maximum fetal heart rate of 220 beats/min. Fifteen minutes after birth, the neonate was admitted to the pediatric intensive care unit for tachycardia and respiratory distress. On admission, vital signs were as follows: temperature was 36.5 ℃, heart rate was 225 beats/min, respiratory rate was 50 breaths/min, and peripheral oxygen saturation was 95% on nasal cannula oxygen. He appeared pale, with grunting respirations, chest retractions, cold extremities, and a capillary refill time > 3 s. Endotracheal intubation was performed, but his peripheral perfusion showed no significant improvement.

Bedside echocardiography showed a left ventricular ejection fraction of approximately 40%, a collapsed inferior vena cava, and hypotension (37/28 mmHg), consistent with neonatal shock. Epinephrine and normal saline volume expansion were initiated. An umbilical arterial catheter and an umbilical venous catheter were placed to establish vascular access and facilitate hemodynamic monitoring and treatment. Electrocardiography demonstrated supraventricular tachycardia, for which continuous intravenous amiodarone was administered for heart-rate control, together with epinephrine for circulatory support. Mechanical ventilation and pulmonary surfactant were subsequently required for neonatal respiratory distress syndrome and pneumonia. By hospital day 3, sinus rhythm had been restored, the heart rate had decreased to 130 beats/min, peripheral oxygen saturation had stabilized at 96%–100%, and blood pressure had increased to 65/40 mmHg.

On hospital day 4, the left lower limb became discolored and cool, although spontaneous movement remained intact. Local warming produced no improvement. Blood pressure in the left leg was 51/31 mmHg, compared with 95/46 mmHg in the right leg and 85/57 mmHg in the right arm. Emergency Doppler ultrasonography demonstrated two contiguous thrombi extending from the proximal left external iliac artery to the femoral artery, measuring 21 × 1.2 mm and 21 × 2.5 mm, with associated vascular filling defects. Left external iliac–femoral arterial thrombosis was diagnosed. Given its temporal relationship to umbilical arterial catheterization and the concordant anatomical distribution, the event was considered catheter-associated (8).

Because the thrombosis was localized, distal limb movement was preserved, and invasive thrombectomy in a neonate carried a substantial risk of vascular injury, pharmacological thrombolysis was selected. Intravenous urokinase was administered for 30 h using a stepwise dose-reduction regimen: 7,200 IU/kg/h for 20 min, 1,800 IU/kg/h for 12 h, 500 IU/kg/h for 6 h, and 400 IU/kg/h for 12 h. Subcutaneous low-molecular-weight heparin (100–150 IU/kg every 12 h) was administered concomitantly and continued after urokinase discontinuation, with the dose adjusted according to serial coagulation monitoring. Limb color, temperature, arterial pulsation, oxygen saturation waveform, and blood pressure progressively normalized. On day 3 after diagnosis, ultrasonography showed that the thrombi had decreased in size to 15 × 1.2 mm and 16 × 2.1 mm, respectively. Complete thrombus resolution was documented on day 6 after diagnosis, at which time low-molecular-weight heparin was discontinued. Oral rivaroxaban at 0.9 mg every 8 h was initiated 2 h before discontinuation of low-molecular-weight heparin to provide uninterrupted anticoagulation. No clinically evident major bleeding or recurrent limb ischemia was observed during the subsequent 3-month follow-up period.

### 2.2 Case 2: postoperative intracranial large-vessel occlusion

A 5-year-and-5-month-old boy with prenatally diagnosed pulmonary sequestration underwent elective thoracoscopic resection. The procedure was uneventful, and his initial postoperative recovery was satisfactory. On postoperative day 3, he suddenly developed lethargy and reduced responsiveness. When he cried, the left corner of his mouth deviated to the left. Motor activity of the left extremities was preserved, whereas muscle strength, assessed using the Medical Research Council scale, was graded as 1/5 in the right upper extremity and 2/5 in the right lower extremity. The right Babinski sign was positive. Emergency brain magnetic resonance imaging (MRI) and magnetic resonance angiography (MRA) demonstrated complete arterial occlusion extending from the left internal carotid artery to the main trunk of the left middle cerebral artery, accompanied by extensive acute ischemic infarction involving the left frontal, parietal, temporal, and insular regions, as well as the basal ganglia and hippocampus (Figures 1A–C).

**Figure 1:**
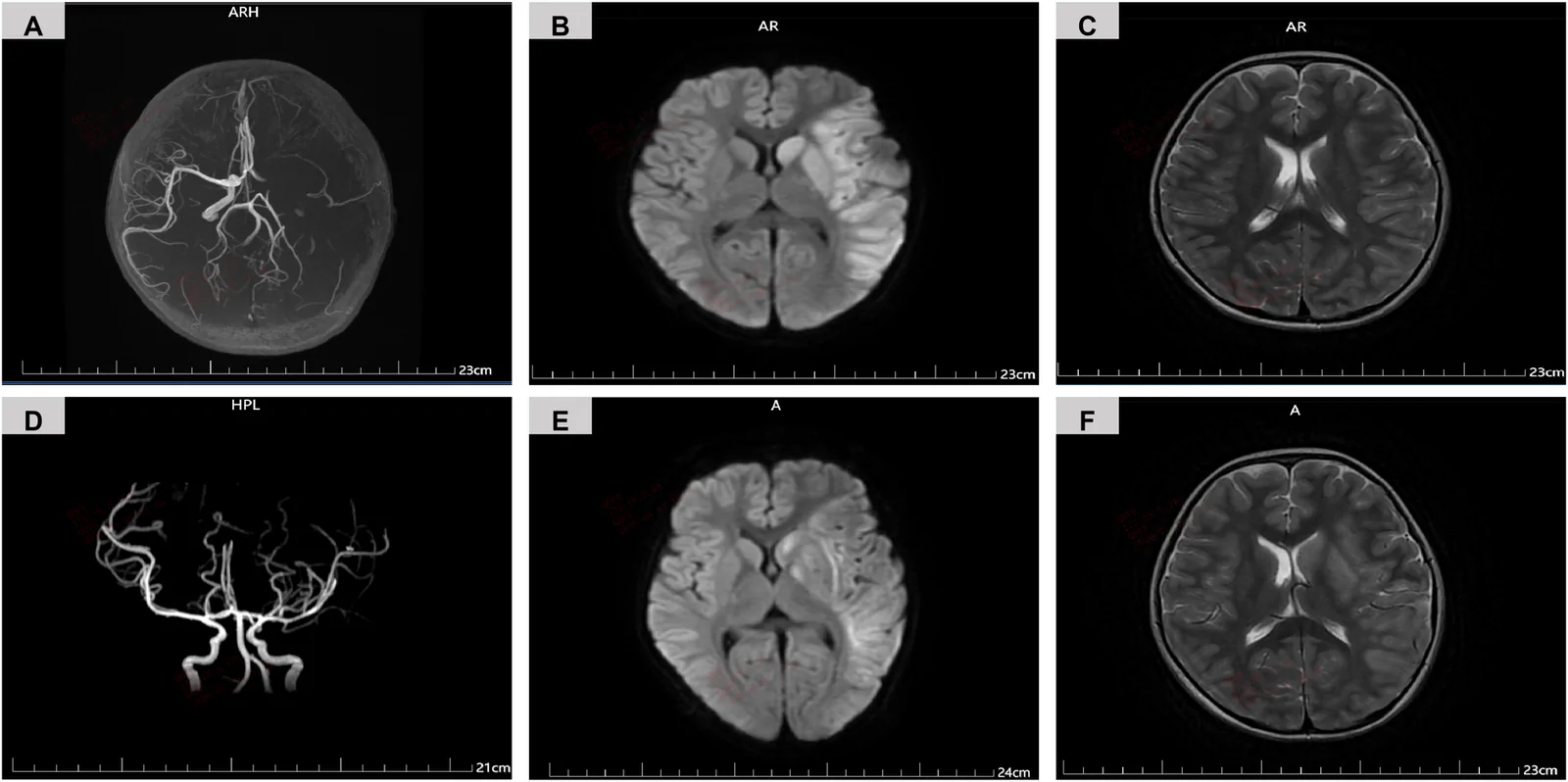
Neuroimaging findings before and one week after mechanical thrombectomy in case 2. **(A–C)** Neuroimaging obtained at the diagnosis of acute ischemic stroke. Magnetic resonance angiography (MRA) demonstrates complete occlusion extending from the left internal carotid artery to the main trunk of the left middle cerebral artery **(A)** Representative axial diffusion-weighted imaging (DWI) **(B)** and T2-weighted imaging **(C)** show extensive acute ischemic infarction involving the left frontal, parietal, temporal, and insular regions, as well as the basal ganglia and hippocampus. **(D–F)** Follow-up neuroimaging obtained one week after mechanical thrombectomy. MRA demonstrates sustained patency of the previously occluded vessels and restoration of intracranial blood flow **(D)** Follow-up axial DWI **(E)** and T2-weighted imaging **(F)**, shown at levels matched to B and C, respectively, show interval evolution of the cerebral infarction.

Given the disabling neurological deficits and the presence of a proximal large-vessel occlusion, emergency cerebral angiography and endovascular treatment were performed under general anesthesia. Angiography demonstrated occlusion of the left internal carotid artery distal to the ophthalmic segment, with non-visualization of the left middle cerebral artery. Mechanical thrombectomy was performed using a stent retriever combined with aspiration. Immediate postprocedural angiography demonstrated complete recanalization and restoration of cerebral perfusion. Follow-up MRI and MRA performed one week after thrombectomy confirmed sustained patency of the previously occluded vessels and showed interval evolution of the cerebral infarction (Figures 1D–F). Following rehabilitation, the child was discharged 38 days after thrombectomy. At discharge, he was able to walk independently with a near-normal gait, although mild involuntary movements of the right upper extremity and slight adduction of the right thumb persisted.

### 2.3 Case 3: cerebral infarction after aortic balloon angioplasty

A 3-month-and-30-day-old boy with prior repair of aortic arch interruption and patent ductus arteriosus ligation was admitted for recurrent coarctation. Aortography showed an isthmic diameter of 1.6 mm and a 70-mmHg systolic gradient, which was reduced to 7 mmHg after balloon angioplasty.

On the first day after balloon angioplasty, he developed paroxysmal twitching of the left eyelid and left upper extremity. CT showed a poorly defined right frontal hypodensity with adjacent sulcal effacement. MRI identified a 28 × 51 mm acute ischemic lesion involving the right frontotemporal and insular cortex and subcortical white matter, with high diffusion-weighted imaging (DWI) signal, low apparent diffusion coefficient signal, and local edema. MRA showed fewer right middle cerebral artery branches in the affected region (Figures 2A,B). No intracranial large-vessel trunk occlusion was identified.

**Figure 2:**
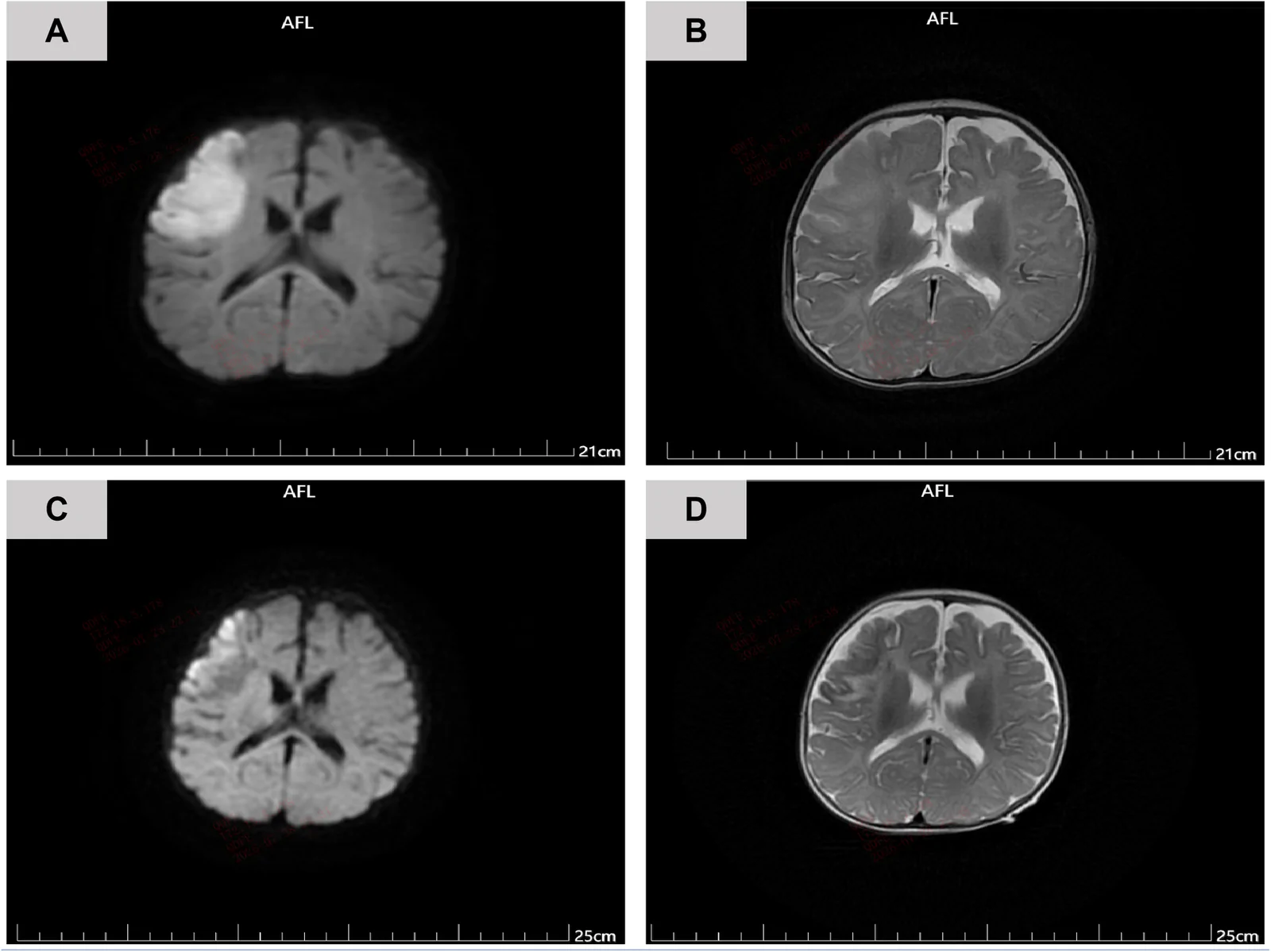
Serial brain MRI findings at diagnosis and one-week follow-up in Case 3. **(A**, **B)** Brain MRI obtained at the diagnosis of cerebral infarction. Axial diffusion-weighted imaging (DWI) shows marked hyperintensity in the right frontotemporal and insular regions, consistent with acute ischemic infarction **(A)**, with a corresponding signal abnormality on T2-weighted imaging **(B)**. **(C**, **D)** Follow-up MRI obtained one week after diagnosis. Axial DWI **(C)** and T2-weighted imaging **(D)** demonstrate decreased DWI signal intensity and a reduction in the extent of the original right frontotemporal–insular lesion. A new patchy area of mild DWI hyperintensity with indistinct margins is visible in the right thalamus, raising suspicion of a new acute ischemic lesion.

Recent cardiac intervention and incompletely healed surgical wounds raised concern about bleeding, while the absence of a retrievable large-vessel occlusion argued against mechanical thrombectomy. Aspirin and low-molecular-weight heparin were administered with mannitol, intravenous phenobarbital, and oral levetiracetam. Follow-up MRI performed one week after the diagnosis of cerebral infarction demonstrated decreased DWI signal intensity and a reduced extent of the original right frontotemporal–insular lesion. Meanwhile, a newly developed patchy lesion with subtle DWI hyperintensity and ill-defined margins was identified in the right thalamus, raising suspicion of an acute infarct (Figures 2C,D). No further limb twitching occurred, and muscle strength and motor function subsequently recovered without a clinically evident residual neurological deficit.

## 3 Discussion

The reported frequency of pediatric arterial thrombosis varies according to the population, preceding procedure, catheter type, screening method, and event definition. In a prospective study of 615 indwelling arterial catheters, thrombosis occurred after 20 catheter insertions (3.25%); 18 events occurred among 137 femoral arterial catheters (13.1%). Younger age, lower body weight, low cardiac output, and increased hematocrit were associated with femoral arterial thrombosis (9). A systematic review reported a pooled cumulative incidence of catheter-related arterial thrombosis of 21% (95% CI, 13%–31), including 20% for umbilical arterial catheters and 11% for cardiac catheter-related events (10). These procedure-specific estimates provide relevant context for Case 1 but cannot be applied directly to perioperative cerebral arterial ischemic stroke or to the general pediatric population.

The three patients had different but overlapping risk profiles. In Case 1, neonatal age, shock and low cardiac output, respiratory distress syndrome, pneumonia, and umbilical arterial catheterization coincided with external iliac–femoral arterial thrombosis. In Case 2, intracranial large-vessel occlusion occurred three days after thoracoscopic surgery. In Case 3, congenital heart disease, previous cardiac surgery, recurrent aortic coarctation, and recent balloon angioplasty were plausible contributors. These features represent documented exposures or potential risk factors rather than proof of a specific thromboembolic mechanism. Multidisciplinary etiological assessment is therefore important and should incorporate cardiac evaluation, vascular imaging, infection assessment, and coagulation testing when clinically indicated.

In Case 1, the limb remained viable and the thrombi were localized, creating an opportunity for pharmacological treatment under serial clinical, laboratory, and ultrasonographic monitoring. Management required integration of the critical care assessment of limb viability and bleeding risk, the ultrasonographic definition and surveillance of the arterial obstruction, and hematological guidance on thrombolytic and anticoagulant treatment. Small neonatal vessels make invasive thrombectomy technically challenging and increase the risks of dissection, perforation, and distal embolization. A cohort of 236 children with iliofemoral arterial thrombosis found that heparin followed by enoxaparin and enoxaparin monotherapy were the most common regimens, whereas only 3.4% underwent surgery (11). Another series of 151 children with acute limb ischemia reported that 91% of events were catheter-related, 94% were managed nonoperatively, and 15% developed complications related to ischemia or treatment during follow-up (12).

A systematic review found that thrombolysis, heparin, and thrombectomy had all been used, alone or in combination, with complete thrombus resolution reported in 82%; however, the evidence was heterogeneous and predominantly observational (10). In our patient, the multidisciplinary assessment favored pharmacological treatment because spontaneous limb movement was preserved, tissue loss was not imminent, and invasive intervention carried substantial risk in a neonate. Intravenous urokinase and low-molecular-weight heparin were followed by complete ultrasonographic resolution six days after diagnosis. No clinically evident major bleeding or recurrent limb ischemia was observed during the 3-month follow-up period. This result supports the feasibility of closely monitored pharmacological treatment but does not establish its superiority over anticoagulation alone or other interventions.

Case 2 represented a different emergency. The child had disabling neurological deficits and complete occlusion extending from the left internal carotid artery to the main trunk of the left middle cerebral artery. Rapid collaboration among pediatric critical care, neurology, neuroradiology, neurointervention, and anesthesiology was required to interpret the neurological findings, confirm the proximal large-vessel occlusion, evaluate procedural feasibility, and proceed with emergency reperfusion. The visible endovascular target, severe deficit, and risk of irreversible cerebral injury favored mechanical thrombectomy despite the limited pediatric evidence.

Available evidence for pediatric thrombectomy remains largely observational. A multi-institutional study reported successful reperfusion in 83% of 23 thrombectomy procedures, with 86% of patients achieving a modified Rankin Scale score of 0–2 at 90 days (13). A recent meta-analysis reported successful recanalization in 88%, favorable functional outcomes in 80%, symptomatic intracranial hemorrhage in 2%, and mortality in 4%, although study heterogeneity and selection bias limit interpretation (14). In our patient, stent-retriever thrombectomy combined with aspiration achieved immediate recanalization, and vessel patency was maintained on one-week MRA. Subsequent rehabilitation formed an additional component of multidisciplinary care. The child regained independent ambulation, although mild involuntary movements of the right upper extremity and slight right thumb adduction remained at discharge. This outcome demonstrates technical feasibility in this selected patient but cannot establish the general efficacy or safety of thrombectomy in children.

In Case 3, the treatment decision required joint consideration of the cardiac intervention, cerebral imaging findings, bleeding risk, and absence of an endovascular target. No retrievable intracranial large-vessel occlusion was identified, while the recent balloon angioplasty and incompletely healed surgical wounds increased concern about bleeding. After multidisciplinary assessment involving cardiology, critical care, neurology, imaging, and hematology, medical management with aspirin, low-molecular-weight heparin, antiseizure therapy, and supportive treatment was selected.

Follow-up MRI showed regression of the initial right frontotemporal–insular lesion but revealed a new patchy area of mild DWI hyperintensity in the right thalamus. Because corresponding ADC restriction was not clearly documented, this finding was considered suspicious for a new acute ischemic lesion rather than diagnostic of recurrent infarction; peri-ictal change and T2 shine-through remain alternative explanations. This uncertainty illustrates the value of joint neurological and radiological reassessment. It also cautions against interpreting clinical improvement alone as evidence of complete radiological stabilization and supports continued neurological surveillance and follow-up imaging after pediatric cardiac intervention.

Across the three cases, multidisciplinary care served different but complementary functions. First, rapid bedside recognition by the primary and critical care teams triggered vascular or cerebral imaging. Second, radiologists and ultrasonographers defined the vascular territory, thrombus burden, and presence or absence of an intervention target. Third, hematology, cardiology, neurology, surgery, and interventional specialists jointly weighed tissue viability, potential benefit of reperfusion, bleeding risk, patient size, and procedural feasibility. Finally, repeated imaging, anticoagulation or thrombolysis monitoring, neurological assessment, and rehabilitation were used to evaluate treatment response and residual deficits. Thus, multidisciplinary management did not imply the use of the same treatment pathway for every patient; it enabled selection of a treatment suited to each child's anatomical and clinical circumstances.

A practical decision framework can therefore be proposed. The first step is to determine whether limb, cerebral tissue, or another organ is immediately threatened. The vascular territory and anatomy should then be defined, including whether the event involves a peripheral artery, a retrievable intracranial large-vessel occlusion, or smaller cerebral branches without an endovascular target. Treatment decisions should subsequently integrate patient age and size, symptom severity, time from onset, thrombus location and burden, tissue viability, recent surgery or intervention, bleeding risk, and local expertise. Anticoagulation may prevent thrombus propagation but may not restore perfusion rapidly; thrombolysis can promote thrombus resolution but increases bleeding concern; and surgical or endovascular thrombectomy may provide rapid reperfusion when tissue is threatened and an accessible target is present. Current evidence does not establish a universally preferred strategy for pediatric arterial thrombosis (5, 15).

This report has several limitations. It is a retrospective description of only three patients with heterogeneous vascular events, and treatment selection was neither standardized nor randomized. The series lacks a denominator of all arterial catheterizations, operations, and cardiac interventions performed during the study period and therefore cannot estimate institutional incidence. Etiological investigations, follow-up duration, and functional outcome assessments were not uniform, and the right thalamic lesion in Case 3 was not definitively characterized. Some decisions may also have been influenced by local expertise and resource availability. Consequently, these observations cannot establish comparative effectiveness or quantify treatment safety. They instead illustrate how prompt anatomical diagnosis and multidisciplinary, risk-adapted decision-making may guide individualized treatment of pediatric arterial thrombosis and ischemic stroke.

## 4 Conclusion

Pediatric arterial thromboembolism requires rapid anatomical definition and individualized risk assessment. In this series, localized neonatal peripheral thrombosis was managed pharmacologically, disabling intracranial large-vessel occlusion prompted emergency thrombectomy, and postoperative cerebral infarction without a large-vessel target was managed conservatively because of bleeding risk. These observations are hypothesis-generating and support a structured multidisciplinary approach; they do not establish the superiority, efficacy, or safety of any strategy.

## 5 Patient perspective

A formal patient or guardian perspective was not collected. The parents or legal guardians were informed of the clinical course and provided written consent for publication of the clinical details and images.

## Data Availability

The raw data supporting the conclusions of this article will be made available by the authors, without undue reservation.
